# Identification of loci of functional relevance to Barrett’s esophagus and esophageal adenocarcinoma: Cross-referencing of expression quantitative trait loci data from disease-relevant tissues with genetic association data

**DOI:** 10.1371/journal.pone.0227072

**Published:** 2019-12-31

**Authors:** Julia Schröder, Vitalia Schüller, Andrea May, Christian Gerges, Mario Anders, Jessica Becker, Timo Hess, Nicole Kreuser, René Thieme, Kerstin U. Ludwig, Tania Noder, Marino Venerito, Lothar Veits, Thomas Schmidt, Claudia Fuchs, Jakob R. Izbicki, Arnulf H. Hölscher, Dani Dakkak, Boris Jansen-Winkeln, Yusef Moulla, Orestis Lyros, Stefan Niebisch, Matthias Mehdorn, Hauke Lang, Dietmar Lorenz, Brigitte Schumacher, Rupert Mayershofer, Yogesh Vashist, Katja Ott, Michael Vieth, Josef Weismüller, Elisabeth Mangold, Markus M. Nöthen, Susanne Moebus, Michael Knapp, Horst Neuhaus, Thomas Rösch, Christian Ell, Ines Gockel, Johannes Schumacher, Anne C. Böhmer

**Affiliations:** 1 Institute of Human Genetics, University of Bonn, School of Medicine & University Hospital Bonn, Bonn, Germany; 2 Institute for Medical Biometry, Informatics, and Epidemiology, University of Bonn, School of Medicine & University Hospital Bonn, Bonn, Germany; 3 Department of Medicine II, Sana Klinikum, Offenbach, Germany; 4 Department of Internal Medicine II, Evangelisches Krankenhaus, Düsseldorf, Germany; 5 Department of Interdisciplinary Endoscopy, University Hospital Hamburg-Eppendorf, Hamburg, Germany; 6 Department of Gastroenterology and Interdisciplinary Endoscopy, Vivantes Wenckebach-Klinikum, Berlin, Germany; 7 Center for Human Genetics, University Hospital Marburg, Marburg, Germany; 8 Department of Visceral, Transplant, Thoracic and Vascular Surgery, University Hospital of Leipzig, Leipzig, Germany; 9 Department of Gastroenterology, Hepatology and Infectious Diseases, Otto-von-Guericke University Hospital, Magdeburg, Germany; 10 Institute of Pathology, Klinikum Bayreuth, Bayreuth, Germany; 11 Department of General, Visceral and Transplantation Surgery, University of Heidelberg, Heidelberg, Germany; 12 Department of General, Visceral, and Cancer Surgery, University of Cologne, Cologne, Germany; 13 Department of General, Visceral, and Thoracic Surgery, University Medical Center Hamburg-Eppendorf, University of Hamburg, Hamburg, Germany; 14 Department of Internal Medicine and Gastroenterology, Elisabeth Hospital, Essen, Germany; 15 Department of General, Visceral, and Transplant Surgery, University Medical Center, University of Mainz, Mainz, Germany; 16 Department of General, Visceral, and Thoracic Surgery, Klinikum Darmstadt, Darmstadt, Germany; 17 Gastroenterologie am Burgweiher, Bonn, Germany; 18 Kantonsspital Aarau, Aarau, Switzerland; 19 Department of General, Visceral, and Thorax Surgery, RoMed Klinikum Rosenheim, Rosenheim, Germany; 20 Gastroenterologische Gemeinschaftspraxis, Koblenz, Germany; 21 Centre of Urban Epidemiology, Institute of Medical Informatics, Biometry, and Epidemiology, University of Essen, Essen, Germany; Kunming Institute of Zoology, Chinese Academy of Sciences, CHINA

## Abstract

Esophageal adenocarcinoma (EA) and its precancerous condition Barrett’s esophagus (BE) are multifactorial diseases with rising prevalence rates in Western populations. A recent meta-analysis of genome-wide association studies (GWAS) data identified 14 BE/EA risk loci located in non-coding genomic regions. Knowledge about the impact of non-coding variation on disease pathology is incomplete and needs further investigation. The aim of the present study was (i) to identify candidate genes of functional relevance to BE/EA at known risk loci and (ii) to find novel risk loci among the suggestively associated variants through the integration of expression quantitative trait loci (eQTL) and genetic association data. eQTL data from two BE/EA-relevant tissues (esophageal mucosa and gastroesophageal junction) generated within the context of the GTEx project were cross-referenced with the GWAS meta-analysis data. Variants representing an eQTL in at least one of the two tissues were categorized into genome-wide significant loci (P < 5×10^−8^) and novel candidate loci (5×10^−8^ ≤ P ≤ 5×10^−5^). To follow up these novel candidate loci, a genetic association study was performed in a replication cohort comprising 1,993 cases and 967 controls followed by a combined analysis with the GWAS meta-analysis data. The cross-referencing of eQTL and genetic data yielded 2,180 variants that represented 25 loci. Among the previously reported genome-wide significant loci, 22 eQTLs were identified in esophageal mucosa and/or gastroesophageal junction tissue. The regulated genes, most of which have not been linked to BE/EA etiology so far, included *C2orf43/LDAH*, *ZFP57*, and *SLC9A3*. Among the novel candidate loci, replication was achieved for two variants (rs7754014, P_combined_ = 3.16×10^−7^ and rs1540, P_combined_ = 4.16×10^−6^) which represent eQTLs for *CFDP1* and *SLC22A3*, respectively. In summary, the present approach identified candidate genes whose expression was regulated by risk variants in disease-relevant tissues. These findings may facilitate the elucidation of BE/EA pathophysiology.

## Introduction

Esophageal adenocarcinoma (EA) represents one of the most rapidly increasing cancers in Western populations [1]. Despite new treatment strategies, mortality rates among EA patients remain high [1]. EA is preceded by the precancerous condition Barrett’s esophagus (BE), which is characterized by a metaplastic transformation of the squamous epithelium in the distal esophagus. Here, the normal stratified squamous epithelium at the gastroesophageal junction is replaced by columnar epithelium, commonly found in the lower gastrointestinal tract. The prevalence of BE in the general population of Western countries is 1.6% [2]. Reported non-genetic risk factors for BE/EA include gastroesophageal reflux, obesity, and age > 50 years [3]. Additionally, family studies of EA and BE have implicated genetic factors in disease development and progression, thus demonstrating that the etiology of BE/EA is multifactorial [4,5]. Furthermore, genetic research has shown that BE and EA display a polygenic overlap [6].

In a recent meta-analysis of data from genome-wide association studies (GWAS), separate (BE and EA) and combined (BE/EA) analyses identified 14 genetic risk loci for BE/EA [7]. The majority of the associated variants map to intergenic or intronic regions of the genome, which renders the identification of the disease-relevant genes and underlying pathomechanisms difficult. Since many non-coding GWAS risk variants exert their effects via gene regulatory mechanisms, expression quantitative trait loci (eQTL) analyses make an important contribution to the elucidation of multifactorial disease etiology [8,9]. In eQTL studies, the alleles or genotypes of genetic variants are correlated with the quantitative expression level of transcripts [10], thereby identifying genetic variants that influence the expression level of a gene. This method is useful for identification of candidate genes at risk loci for functional follow-up studies [11–13].

The aim of the present study was (i) to identify novel candidate genes of functional relevance to BE/EA at known risk loci and (ii) to find novel risk loci among the suggestively associated variants. This was accomplished by integrating eQTL data from BE/EA-relevant tissues (esophageal mucosa and gastroesophageal junction) [14] and genetic data from the recent BE/EA GWAS meta-analysis [7]. Variants with suggestive evidence for association were further investigated in a genetic association analysis in an independent replication case-control cohort. To increase statistical power, association data were combined with the data of the previous GWAS meta-analysis [7].

## Materials and methods

**Fig 1:**
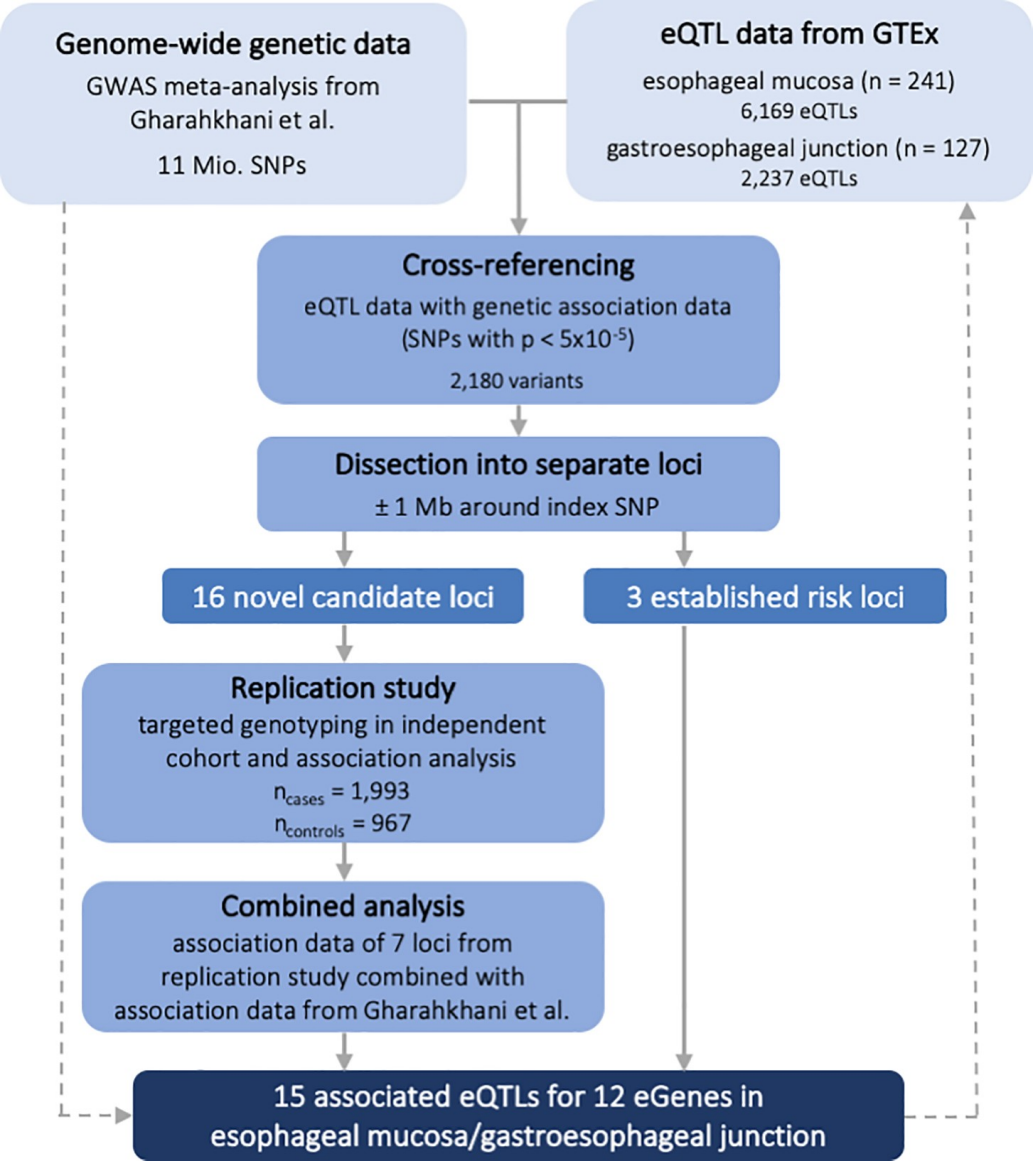
Study workflow.

The study workflow is illustrated in Fig 1.

### Cross-referencing of eQTLs from BE/EA-relevant tissues with BE/EA genetic association data

The GTEx project [14] represents the largest eQTL database to date comprising 152,869 cis-eQTLs from 44 tissues (V6P). Tissue was sampled from donors post-mortem and eQTLs were mapped using tissue-specific RNA sequencing data and genotype data of DNA from whole blood. Two tissue types most relevant to BE/EA were selected from the GTEx database [14]: esophageal mucosa and gastroesophageal junction. Both datasets were restricted to eQTLs with a false discovery rate (FDR) of < 0.05. The esophageal mucosa sample comprised tissues of 241 individuals with 6,169 cis-eQTLs (eQTL-gene located < 1 Mb distance from genetic variant) and the gastroesophageal junction sample comprised tissues of 127 individuals with 2,237 cis-eQTLs.

The eQTLs were cross-referenced to variants that showed at least a suggestive association to BE/EA (P ≤ 5×10^−05^) in the BE/EA GWAS meta-analysis [7]. All variants showing an eQTL effect in one or both tissues were then assigned to distinct genomic loci: The index SNP of each locus was specified as the variant with the most significant BE/EA association and each locus was defined at ± 1 Mb around this variant. All loci on the same chromosome where reviewed for independence by analysis of linkage disequilibrium (LD). Where applicable, long-range LD was taken into account and the respective loci were marked accordingly. We tested the option of applying statistical colocalization analyses on the selected loci using COLOC [15] but found that the analysis was severely underpowered most likely due to a small sample size in the eQTL datasets. The resulting loci were categorized into genome-wide significant loci (P < 5×10^−8^) and novel candidate loci (5×10^−8^ < P ≤ 5×10^−5^). The index SNPs of these candidate loci were then included in the subsequent genetic replication study in an independent BE/EA case-control.

### Replication sample

The case-control cohort for the replication study comprised: (i) 1,117 BE cases and 876 EA cases (total of 1,993 BE/EA patients); and (ii) 967 controls. The cases were recruited in an ongoing effort as described for the Bonn sample in Gharahkhani et al. [7]. All samples included in this replication cohort have not been part of the prior Gharahkhani et al. [7] GWAS and were recruited between November 2014 and February 2018. Patients with suspected BE/EA were recruited in hospitals and clinics where they underwent endoscopies or surgeries. Exclusion criteria were (i) a negative histopathological diagnosis that did not confirm BE/EA disease status and (ii) self-reported descent was non-European. The patients were recruited at 15 medical centers across Germany and blood samples were collected at the University Hospital Leipzig before being sent to Bonn for DNA extraction and genotyping. The control cohort was recruited at the University Hospital Bonn from blood donors, also of European descent. Relevant demographic details for both cohorts can be found in S1 Table.

The study was approved by the ethics committees of the Universities of Bonn and Leipzig. All participants provided written informed consent prior to inclusion.

### Genotyping

Genotyping was performed using the multiplex MALDI-TOF mass spectrometer MassArray system by Agena (San Diego, USA). Of the 16 index SNPs representing the novel candidate loci, three variants (rs59341339, rs11145842, rs12985299) were excluded from the plex for technical reasons. No alternative SNP in high LD was found among the associated eQTLs, and thus the corresponding loci were excluded from the analyses. The index SNPs of three further loci, which were excluded due to the same technical reasons, were replaced by variants in high LD [r^2^ > 0.95; rs2442722 (P = 1.22×10^−6^) was replaced by rs36057735 (P = 5.13×10^−6^), rs76510925 (P = 7.86×10^−6^) by rs12112778 (P = 1.57×10^−5^), and rs11169302 (P = 1.05×10^−5^) by rs9364 (P = 2.23×10^−5^)]. Thus, a total of 13 variants were genotyped in the 1,993 BE/EA cases and 967 controls. Primers for amplification and genotyping were synthesized by Metabion (Martinsried, Germany). For the purposes of quality control (QC), negative controls (H_2_O) and intra- and inter-plate duplicates were added to each plate. After genotyping, clusters were visually inspected, and re-clustering was performed if necessary. Finally, genotype and SNP information files were extracted for the subsequent genetic association analysis.

### Association analyses

Genotype QC and association calculations were performed using R and PLINK [16,17]. SNPs or samples were excluded on the basis of: (i) low call rate (SNPs: ≤ 95%, samples: > 1 missing SNP call); (ii) deviation from Hardy-Weinberg equilibrium (HWE; P < 0.05 in controls). For the replication study, association was calculated using the one-sided Cochran-Armitage trend test in the direction of effect established through the previous GWAS meta-analysis [7]. The effect size was estimated using logistic regression. Standard errors of the effect sizes were calculated with 95% confidence intervals.

All BE/EA association results from the replication sample were then combined with the association results from the BE/EA GWAS meta-analysis [7]. This was performed via a fixed-effect meta-analysis, as based on the standard-error in METAL (version 2011-03-25) [18].

### Downstream analyses

Downstream analyses of the target genes of the reported variants were performed using the tool STRING [19]. The STRING database is a collection of protein-protein interaction information that also integrates tools for pathway analyses such as Gene Ontology and KEGG. A gene-set enrichment analysis was performed on the list of target genes (see Tables 1 and 2) and analyzed for possible protein-protein interactions and enrichment in pathways.

**Table 1:** Genome-wide significant loci resulting from the cross-referencing of eQTL and genetic data.

SNP information				Gharahkhani et al.		GTEx eQTL			
SNP	Chromosome	Position	Alleles ^a^	P-value	Effect	Tissue	eGene	P-value	Effect
**rs7255**	2	20,878,820	T/C	9.12×10^−11^	0.127	Gastro	*C2orf43*	2.54×10^−7^	0.471
						Mucosa	*C2orf43*	6.75×10^−16^	0.479
**rs147462972^b^**	5	622,869	AC/A	3.23×10^−9^	-0.139	Mucosa	*AC026740*.*1*	5.84×10^−11^	-0.547
						Mucosa	*SLC9A3*	3.39×10^−5^	0.362
**rs13220495^c^**	6	26,441,640	C/T	5.36×10^−7^	-0.166	Gastro	*BTN3A2*	9.22×10^−17^	-1.151
						Mucosa	*BTN3A2*	2.05×10^−32^	-1.263
**rs13201294^c^**	6	27,556,141	A/T	2.98×10^−8^	0.169	Gastro	AL022393.7	1.65×10^−5^	0.964
						Mucosa	RP5-874C20.3	1.89×10^−5^	-0.248
						Mucosa	ZSCAN31	2.04×10^−4^	0.373
**rs9257809**	6	29,356,331	A/G	5.93×10^−9^	0.204	Gastro	*ZFP57*	5.24×10^−5^	1.062
						Mucosa	*ZFP57*	4.42×10^−13^	1.368
**rs62413646^b^**	6	58,003,289	A/T	2.58×10^−7^	0.127	Gastro	*LINC00680*	2.02×10^−8^	-0.789
						Mucosa	*LINC00680*	7.58×10^−5^	-0.388
**rs11249893^d^**	8	8,700,851	T/C	7.73×10^−8^	0.102	Mucosa	*FAM86B3P*	5.66×10^−25^	0.762
						Mucosa	*CTA-398F10*.*2*	1.60×10^−9^	0.442
						Mucosa	*ALG1L13P*	1.91×10^−8^	0.505
**rs28630503^b^**	8	10,009,016	T/C	1.20×10^−8^	0.118	Mucosa	*AF131215*.*9*	8.59×10^−7^	0.274
						Mucosa	*AF131215*.*2*	3.03×10^−5^	0.319
**rs10108511**	8	11,435,516	T/C	2.12×10^−9^	0.0188	Mucosa	*AF131215*.*9*	1.63×10^−12^	-0.359
						Mucosa	*AF131215*.*2*	2.88×10^−11^	-0.464
						Mucosa	*FAM167A*	1.26×10^−9^	-0.382
						Mucosa	*RP11-419I17*.*1*	1.29×10^−6^	-0.379

eQTL–expression quantitative trait loci; Gastro–gastroesophageal junction; GTEx–Genotype-Tissue Expression; LD–linkage disequilibrium; Mucosa–esophageal mucosa; SNP–single-nucleotide polymorphism

^a^ Effect allele specified first

^b^ Best-associated SNP at that locus was not present in the GTEx dataset, next best-associated variant was analyzed instead

^c^ Long-range LD with rs9257809, reported in Gharahkhani et al. [7] as single locus

^d^ Long-range LD with rs10108511, reported in Gharahkhani et al. [7] as single locus

**Table 2:** Novel loci resulting from the cross-referencing of eQTL and genetic data.

SNP information				Replication		Combined analysis		GTEx eQTL			
SNP	Chromosome	Position	Alleles^a^	P-value	Effect	P-value	Effect	Tissue	eGene	P-value	Effect
**rs2808207**	6	76,130,215	C/T	0.651	-0.023	1.00×10^−4^	0.074	Mucosa	*SENP6*	5.79×10^−5^	-0.176
**rs7774070**	6	89,911,865	G/A	0.264	0.035	3.31×10^−5^	0.076	Mucosa	*GABRR1*	3.50×10^−5^	-0.358
**rs7754014**	6	160,918,295	T/A	0.028*	0.130	3.16×10^−7^	0.112	Mucosa	*SLC22A3*	6.61×10^−5^	0.322
**rs1626067**	11	67,192,555	A/G	0.926	-0.083	3.10×10^−5^	0.078	Gastro	*PTPRCAP*	8.43×10^−10^	-0.558
								Gastro	*RPS6KB2*	3.95×10^−5^	-0.274
								Mucosa	*PTPRCAP*	9.64×10^−15^	-0.354
**rs9364**	12	50,570,519	G/A	0.544	-0.006	6.41×10^−5^	0.074	Gastro	*LIMA1*	5.21×10^−5^	0.230
**rs1540**	16	75,481,185	C/G	0.019*	0.162	4.16×10^−6^	0.116	Gastro	*CFDP1*	2.32×10^−5^	0.431
**rs1029689**	19	964,051	T/G	0.295	0.042	4.29×10^−5^	0.118	Mucosa	*WDR18*	4.74×10^−9^	-0.594

eQTL–expression quantitative trait loci; Gastro–gastroesophageal junction; GTEx–Genotype-Tissue Expression; Mucosa–esophageal mucosa; SNP–single-nucleotide polymorphism

^a^ Effect allele specified first

* Significant (p < 0.05)

## Results

### Cross-referencing of eQTLs with genetic association data

In total, 6,387 SNPs in the GWAS meta-analysis [7] showed at least a suggestive association with BE/EA (P ≤ 5×10^−5^) and were cross-referenced to the cis-eQTL data from GTEx esophageal mucosa and gastroesophageal junction tissues [14]. Of these, 2,180 SNPs showed eQTL effects in at least one of the two tissues. These variants were assigned to 25 distinct genomic loci (see Materials and Methods, and S2 Table).

Nine of the 25 loci were reported with genome-wide significance by Gharahkhani et al. [7], but we here identified novel downstream target genes at these nine loci based on regulatory effects on gene expression (see Table 1). For three of these loci, the best-associated SNP reported by Gharahkhani et al. [7] was not present in the GTEx dataset, but the locus is represented by the next best SNP (marked with ^b^ in Table 1). Three more loci show long-range LD with another locus and have therefore not been reported as separate loci by Gharahkhani et al. [7] (marked with ^c^ or ^d^ in Table 1).

### Replication of candidate loci

Variants at 16 loci presented both an eQTL effect in relevant tissues and suggestive evidence of association (5x10^-8^ < P ≤ 5x10^-5^) in the GWAS data, respectively. For 13 loci, the index SNP (or a proxy SNP in strong LD) was genotyped in the replication sample. Of the genotyped SNPs, six variants failed QC: five variants showed a call-rate < 95% and another SNP deviated from HWE (P < 0.05 in controls). In addition, 53 samples (32 cases, 21 controls) were excluded due to of the presence of > 1 missing genotype. Details of the final BE/EA replication analysis are shown in Table 2.

Upon statistical analysis, the variant rs1540 on 16q23 showed a nominally significant association to BE/EA in the independent replication study (P_replication_ = 0.019). In the combined analysis, a lower P-value was observed as compared to the meta-analysis data alone (P_meta-analysis_ = 3.02×10^−5^, P_combined_ = 4.16×10^−6^). According to the GTEx data, this variant represents an eQTL for *CFDP1* in gastroesophageal junction tissue (P = 2.32×10^−5^). Here, the BE/EA risk allele leads to an increase in gene expression. Similarly, rs7754014 on 6q25 showed a nominally significant association to BE/EA in the replication study (P_replication_ = 0.028) and a lower P-value in the combined analysis (P_meta-analysis_ = 2.07×10^−6^, P_combined_ = 3.16×10^−7^). According to the GTEx data, this variant represents an eQTL for *SLC22A3* in esophageal mucosa tissue (P = 6.61×10^−5^). Again, the BE/EA risk allele leads to an increase in gene expression.

### Downstream analyses

The target genes of the index variants of the nine genome-wide significant and seven candidate loci were analyzed using STRING. Several genes could not be included in the analyses because they do not code for proteins (RNA genes, pseudogenes). The protein-protein interaction (PPI) analysis of the remaining 14 genes did not show any interactions between the proteins encoded by genes (PPI enrichment p = 1, see S1 Fig). Likewise, the pathway analyses did not yield any significant results.

## Discussion

Previous GWAS have identified a total of 14 genetic risk loci for BE/EA [7,20–22]. However, the mechanisms through which these risk variants exert their effects remain unclear. The aim of the present study was (i) to identify candidate genes of functional relevance to BE/EA at known risk loci and (ii) to find novel risk variants among the suggestively associated variants through the integration of eQTL- and genetic association data. Cross-referencing of eQTL data and genetic data from the recent GWAS meta-analysis yielded 2,180 variants at 25 loci (see S2 Table). Of these, nine loci were established BE/EA risk loci from the GWAS meta-analysis and 16 were novel candidate loci.

The replication study yielded two nominally significant BE/EA-associated variants: rs1540 and rs7754014. Variant rs1540 on 16q23 regulates the expression of the gene *CFDP1* (craniofacial development protein 1) in the gastroesophageal junction. The biological function of *CFDP1* remains unclear. However, research suggests that the protein is involved in both the maintenance of higher-order chromatin organization and cell cycle progression [23]. Variant rs7754014 on 6q25 represents an eQTL for the gene *SLC22A3* (solute carrier family 22 member 3) in the esophageal mucosa. *SLC22A3* encodes the protein OCT3 (organic cation transporter 3), which transports endogenous organic cations as well as drugs and toxins [24,25]. Interestingly, *SLC22A3* expression plays a role in other esophageal disorders: downregulation of *SLC22A3* was reported in patients with familial esophageal squamous cell cancer [26]. Previous authors have therefore proposed that suppression of *SLC22A3* may be implicated in the progression of this cancer type [27]. It remains to be shown how these findings relate to the upregulation of *SLC22A3* as it was observed in BE/EA risk allele carriers through our integrative analysis. The independent replication of these two loci gives evidence to their functional relevance for the BE/EA phenotype. This is further supported by the decrease of the P-value after the combined analysis by one order of magnitude. However, since the effect sizes are small, the P-value has not reached genome-wide significance in the combined sample. Larger patient cohorts are warranted to carry these variants over the threshold of genome-wide significance.

Among the established BE/EA risk loci from the GWAS meta-analysis [7], the present analyses identified three eQTLs with a regulating effect on biologically plausible genes. Most of these eQTLs have not been reported previously despite the fact that cross-referencing with eQTL analyses had been performed in the context of the original GWAS meta-analysis [7]. The reason is most likely the use of GTEx version 6 in the analysis by Gharahkhani et al. [7] for the cross-referencing with eQTL data, as opposed to GTEx version 6P used in the present study. While this new dataset does not differ in respect to sample size, it provides new eQTL results due to an improved gene-level annotation. The most significantly associated risk variant from the BE/EA GWAS meta-analysis was rs7255 on 2p24. This is an eQTL for the expression of the gene *C2orf43* in tissue from the esophageal mucosa and the gastroesophageal junction. This gene encodes the protein LDAH (lipid droplet-associated hydrolase), which is a lipid droplet-associated serine lipid hydrolase [28]. The BE/EA risk variant rs92578209 on 6p22 regulates the expression of the gene *ZFP57* (zinc finger protein 57) in both the esophageal mucosa and the gastroesophageal junction. Research has shown that among others, *ZFP57* plays a key role in cell fate decisions during early mouse gastrulation [29]. The third BE/EA risk variant from the GWAS meta-analysis was rs147462972 on 5p15, which represents an eQTL for the expression of *SLC9A3* (solute carrier family 9 member A3) in the esophageal mucosa. The BE/EA risk allele of this variant results in a structural change in the binding sites of the transcription factors CTCF and RAD21. Interestingly, research has demonstrated an enrichment of somatic mutations in the CTCF binding motif in patients with esophageal cancer [30]. *SLC9A3* encodes the epithelial brush border Na/H-exchanger NHE3, which uses the inward sodium ion gradient to expel acids from the cell [31]. Importantly, an increase in *SLC9A3* expression has been correlated with the severity of gastroesophageal reflux disease, which is a major risk factor for BE [32]. Future studies are warranted to generate further evidence for the involvement of *SLC9A3* in BE/EA development.

The present study had four main limitations. First, the capacity of the GTEx and BE/EA GWAS meta-analysis data to determine whether the eQTLs and BE/EA risk SNPs referred to the same causal variants, or whether they were only correlated, was limited. A different approach using a colocalization analysis could not bring forward significant results due to a lack of power caused by a small sample size of the eQTL samples. The exploratory approach applied in this study may be prone to type I error. Nevertheless, the discovery of genes associated to related phenotypes, such as esophageal squamous cell cancer and GERD, show that our approach has merit. Further research is warranted to establish a causal relationship between these genes and their effect on BE/EA development. Second, the replication sample was too small to achieve a test-wide significant association level in the replication study and a genome-wide significant association level after combination with the previous meta-analysis for the investigated variants. Third, the tissue of origin for development of BE/EA is not completely understood. Several studies discuss the importance of tissue selection in order to detect tissue-specific eQTL effects relevant to disease etiology [33–35]. However, the specific tissue or cell type relevant to a trait or disease is often unknown. In this study, we used eQTL effects in tissues drawn from esophageal mucosa and gastroesophageal junction. Wang [36] discusses the evidence for the squamous epithelium mucosa cell as a precursor for BE/EA, while Zhuang and Fitzgerald [37] debate the existence of a transitional layer at the gastroesophageal junction to be the origin of BE/EA. Thus, to our present knowledge, esophageal mucosa and gastroesophageal junction are the most likely of the currently available tissues to represent the true tissue of origin for BE/EA. Fourth, the highlighted genes have not been yet characterized in functional studies using cellular or animal models. The manner in which the genes are influencing the disease development is currently unclear and requires further investigation.

## Conclusions

Altogether, this study provides a link between BE/EA-associated genetic variants and a regulatory effect on candidate genes in disease-relevant tissues. The present analyses identified biologically plausible candidate genes for BE/EA, such as *SLC22A3* and *SLC9A3*. Notably, *SLC9A3* has already been implicated with gastroesophageal reflux, rendering it an interesting candidate gene. Follow-up analyses are warranted to refine the regulatory annotation and to elucidate the mechanisms through which the implicated variants and genes influence BE/EA development.

## Supporting information

S1 Table Demographic details on replication cohort. (XLSX)

S2 Table 25 risk loci determined after cross-referencing of eQTL and genetic data. (XLSX)

S3 Table Genotype counts of all 13 candidate loci in all analyzed cases and controls. (XLSX)

S1 Fig Results of protein-protein interaction analysis by STRING. (TIFF)

## List of abbreviations

BE: Barrett's esophagus
CFDP1: craniofacial development protein 1
ChIP: chromatin immunoprecipitation
EA: esophageal adenocarcinoma
eQTL: expression quantitative trait locus
FDR: false discovery rate
GTEx: Genotype-Tissue Expression
GWAS: genome-wide association study
HWE: Hardy-Weinberg equilibrium
LD: linkage disequilibrium
LDAH: lipid droplet-associated hydrolase
NHE3: Na/H-exchanger 3
OCT3: organic cation transporter 3
PPI: protein-protein interaction
QC: quality control
SLC22A3: solute carrier family 22 member 3
SLC9A3: solute carrier family 9 member 3
SNP: single-nucleotide polymorphism
ZFP57: zinc finger protein 57
