# Supplementary figures and images for: Identification of loci of functional relevance to Barrett’s esophagus and esophageal adenocarcinoma: Cross-referencing of expression quantitative trait loci data from disease-relevant tissues with genetic association data
Source: PLoS One. 2019 Dec 31;14(12):e0227072. doi: 10.1371/journal.pone.0227072 (PMC6938334; doi:10.1371/journal.pone.0227072)

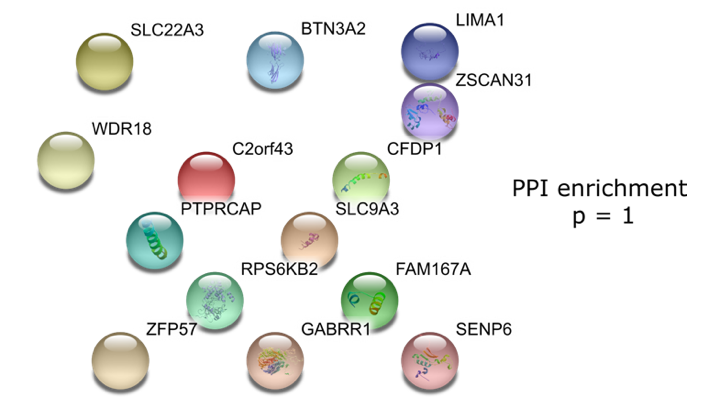

Supplement: S1 Fig — (TIFF) [file pone.0227072.s004.tiff]
